# Superior Vena Cava Syndrome Masquerading as Angioedema: A Chemotherapy Port Complication

**DOI:** 10.7759/cureus.67329

**Published:** 2024-08-20

**Authors:** Philip Mardock, Austin Patrick Eisenberg, Vishal Shah, Thaddeus Golden

**Affiliations:** 1 Internal Medicine, Grand Strand Medical Center, Myrtle Beach, USA; 2 Pulmonary and Critical Care Medicine, Grand Strand Medical Center, Myrtle Beach, USA

**Keywords:** superior vena cava (svc) obstruction, chemotherapy access port, facial angioedema, chemo port, implantable port, superior vena cava (svc), superior vena cava (svc) syndrome

## Abstract

Superior vena cava (SVC) syndrome is a constellation of symptoms that occur secondary to external compression of the SVC, most commonly by a mediastinal malignancy. With the increased use of implanted cardiac devices and indwelling central venous catheters, SVC syndrome from a benign cause has become quite common. This report follows a 62-year-old female who was initially admitted to the critical care unit for treatment of angioedema without a history of malignancy but was found to have a surgically placed port used to treat her rheumatoid arthritis. Despite treatment of what was presumed to be angioedema, her symptoms failed to resolve. Imaging of the thorax revealed a venous thrombosis in the previously placed port. The port was subsequently removed, and the patient's symptoms hastily resolved. This case report underscores the importance of obtaining a thorough history, maintaining a broad differential diagnosis, and revising the differential when the patient’s symptoms fail to improve.

## Introduction

The superior vena cava (SVC) is formed from the unification of the brachiocephalic veins and plays an integral role in venous and lymphatic drainage. Blood from the head, neck, and upper extremities flows through the SVC into the right atrium [1,2]. Occlusion of the SVC forces the body to rely primarily on collaterals to return blood flow. As a result, the severity of SVC symptoms is inversely proportional to the number of developed venous collaterals [3]. SVC syndrome typically presents as neck and facial swelling, dyspnea, and cough that worsens in the supine position. In more severe cases, bilateral proptosis, oropharyngeal edema, or encephalopathy may manifest and require urgent intervention [4].

Approximately 70% of SVC syndrome cases are due to malignant etiologies, such as lung cancers and lymphomas, with the other 30% occurring from intravascular devices, such as pacemakers or permanent venous catheters [4]. Of the malignant etiologies, non-small cell lung cancer comprises about 50%, while small cell lung cancer causes roughly 25% of SVC syndrome [4]. Over the last decade, the placement of permanent indwelling venous catheters and cardiac devices has increased significantly, which has led to increased SVC syndrome in the setting of these devices [5].

A thorough history and physical exam are essential for guiding a diagnosis of SVC syndrome into the list of differential diagnoses. Patients most commonly present with neck or facial swelling, arm swelling, orthopnea, dyspnea, and cough. Less commonly, these patients have dilated chest veins, confusion, and dizziness [6]. SVC syndrome must be confirmed with imaging; a CT scan of the neck/thorax with intravenous contrast has a sensitivity of 96% and a specificity of 92% [7]. Magnetic resonance imaging and ultrasound may be helpful in specific clinical scenarios, such as pregnancy or when CT requiring IV contrast is contraindicated [8]. Still, the speed, sensitivity, and specificity of the CT scan make it an excellent choice for the diagnosis of SVC syndrome [7,8].

Treatment of SVC syndrome depends on the etiology. For malignant etiologies, a biopsy is needed if the histology of the tumor is unknown, as this will guide oncologic treatment, such as chemotherapy versus radiation versus surgical resection. PET-CT scans may also be necessary for staging the malignancy to further assist oncologists in tailoring a treatment regimen [8].Endovascular therapies such as angioplasty, stenting, and thrombolysis have been proven to be effective in managing the symptoms stemming from SVC syndrome. Endovascular therapy tends to have better outcomes when the etiology is benign [9]. If the cause of SVC syndrome is an iatrogenic device, removing the offending component should alleviate symptoms, as seen in this case. Anticoagulation should also be considered in those patients with thrombotic occlusion who do not have a significantly elevated bleeding risk [4].

## Case presentation

This case follows a 62-year-old female who presented to the hospital with complaints of facial swelling, shortness of breath, and stridor. Her medical history includes insulin-dependent diabetes, hypertension, asthma, tracheobronchomalacia, rheumatoid arthritis, and intellectual disability. Additionally, her medication allergy list included lisinopril after multiple prior hospitalizations and at least two intubations in the two months for angioedema before the hospitalization chronicled here. Upon her arrival at the emergency department, she received two doses of epinephrine, 0.3 mg intramuscularly and 125 mg of methylprednisolone intravenously, racemic epinephrine 0.5 mL, budesonide 1 mg, and albuterol with ipratropium via nebulizer. The patient was brought to the intensive care unit for continued monitoring and airway management. She was initially placed on 10 liters of oxygen via nasal cannula with a respiratory rate of 21 breaths per minute due to her respiratory distress. Her heart rate ranged from 90-100 beats per minute, with a blood pressure of 129/67 mmHg. She was afebrile. Her labs upon admission showed hyperglycemia following steroid administration and elevated lactic acid that normalized on a repeat draw. After she received the aforementioned medication regimen, the patient's supplemental oxygen was reduced to 3 liters on a nasal cannula. Once her breathing stabilized, she confirmed that she had continued the use of lisinopril despite her severe reactions and recommendations to discontinue the medication.

With this patient’s presentation, continued use of lisinopril, and previous admissions for angioedema attributed to lisinopril use, her diagnosis appeared evident. However, her physical exam was not consistent with ACE-inhibitor-induced angioedema. Her lungs, on auscultation, demonstrated diffuse wheezes. Auscultation of her upper airway and neck was significant for stridor. Her face was flushed and swollen. However, her oropharynx did not show signs of erythema or edema. Her anterior neck was palpated, and erythema and edema were demonstrated. 

The patient was transported to the critical care unit. Her stridor, wheezing, and swollen presentation improved, and her supplemental oxygen requirements decreased. Due to the patient’s clinical improvement, intubation was deferred.

**Figure 1:**
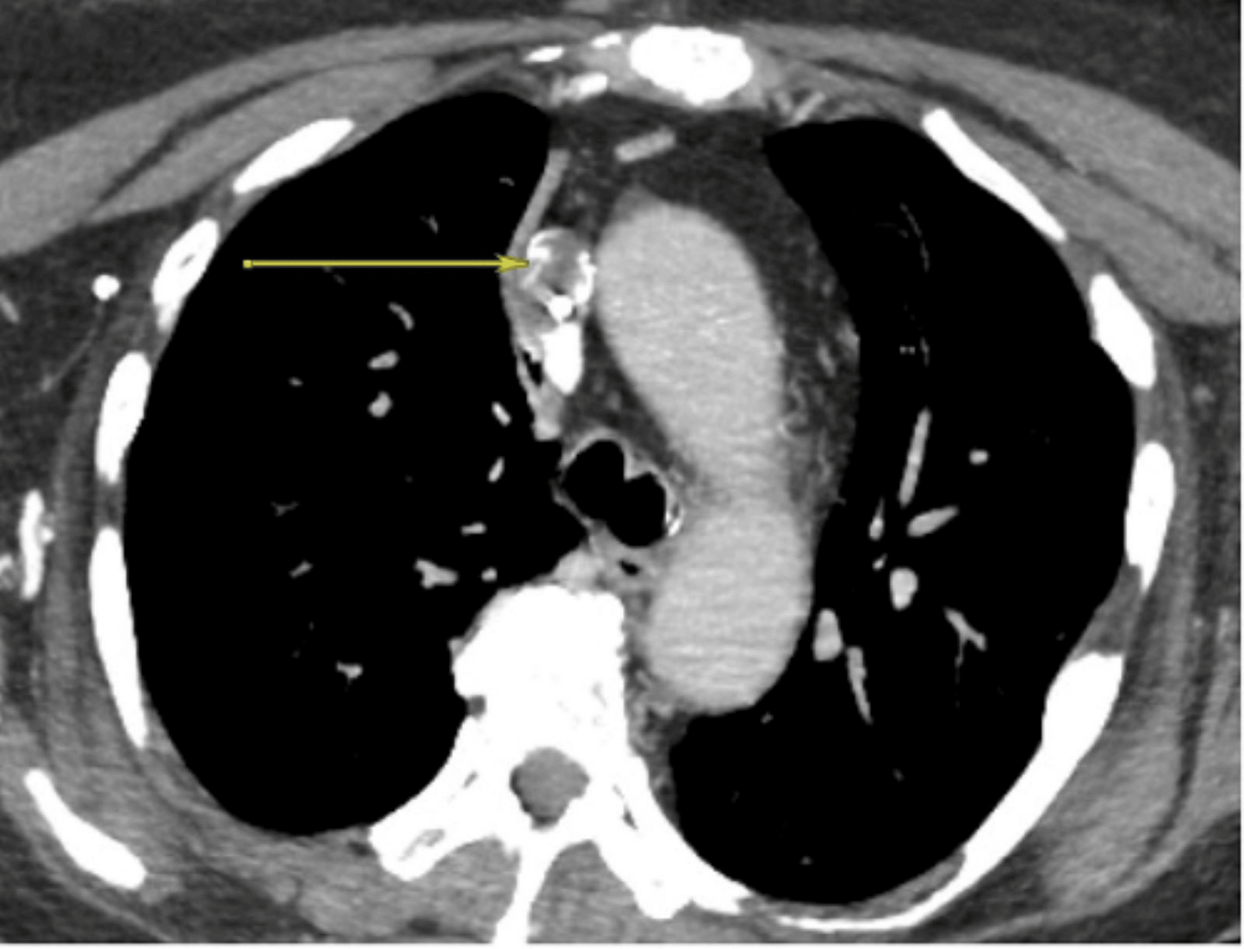
CTA thorax with IV contrast (transverse view) demonstrating a thrombus (yellow arrow) in the SVC, resulting in irregular filling defects. CTA, computed tomography angiography; IV, intravenous; SVC, superior vena cava.

**Figure 2:**
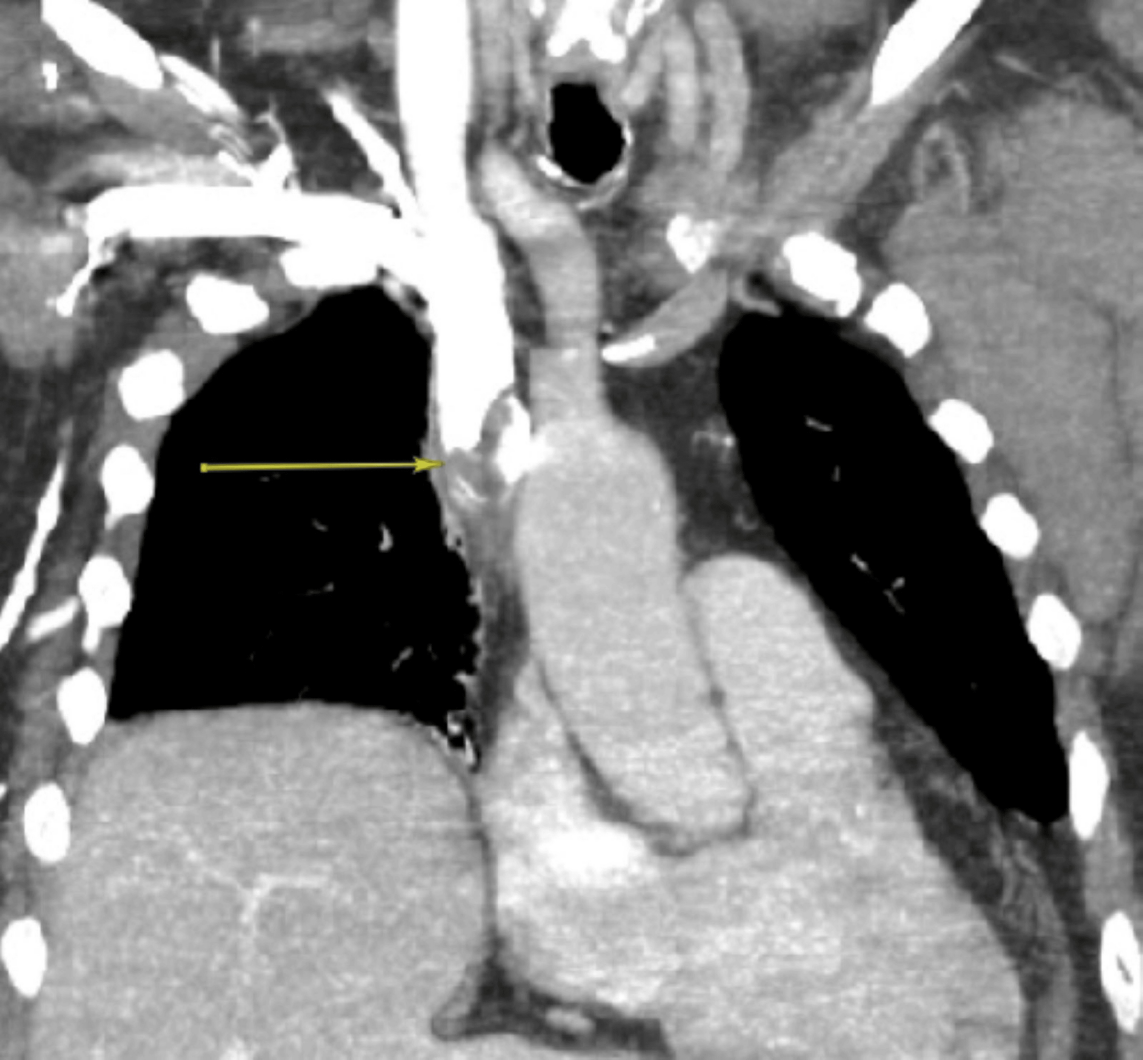
CTA thorax with IV contrast (coronal view) demonstrating catheter-associated thrombosis in the SVC (yellow arrow) suspected with irregular filling defect extending to the superior cavoatrial junction with associated prominent collateral vessels in the right upper mediastinum and supraclavicular fossa. CTA, computed tomography angiography; IV, intravenous; SVC, superior vena cava.

Upon her arrival to the ICU, the patient was further examined and noted to have chemo-port access to the right anterior chest wall, which was placed six years prior for rheumatoid arthritis infusion treatments. A CT scan of the neck and chest with intravenous contrast was obtained to characterize the patient’s airway further. The scan identified an irregular filling defect at the distal aspect of the patient’s right internal jugular-approach port catheter extending to the superior cavoatrial junction with prominent collateral vessels extending into the supraclavicular fossa and the upper mediastinum (Figures 1, 2). With this imaging, the patient’s differential diagnosis was broadened to include SVC syndrome. The interventional radiologists were consulted to assess these new findings.

The patient was taken to the interventional radiology department the following morning for an angiogram with subsequent removal of her port catheter. She also underwent balloon angioplasty of the SVC defect region due to confirmed stenosis and thrombosis. Kissing stents were placed and extended from the bilateral innominate veins to the SVC. After the procedure, the patient was started on apixaban 5 mg twice daily and aspirin 81 mg. Following the procedure, the patient was transferred from the intensive care unit to the medical floor due to her rapid improvement. She was discharged shortly afterward.

## Discussion

The left and right brachiocephalic veins form the SVC, the blood vessel primarily responsible for draining blood from the head, neck, and upper extremities. The SVC can become occluded for a multitude of reasons, most commonly via external compression or thrombosis, leading to a constellation of symptoms deemed SVC syndrome [4].

SVC syndrome commonly presents with symptoms such as headaches, blurry vision, cough, tongue swelling, dyspnea, stridor, and swelling of the face, neck, and upper extremities [1].The onset and severity of symptoms depend on how quickly the SVC becomes occluded and the number of collateral vessels that form [9]. Some of the more severe symptoms of SVC syndrome, such as face and tongue swelling, dyspnea, and stridor, overlap with those of angioedema, making this diagnosis difficult. It was initially thought that our patient was experiencing recurrent angioedema in the setting of lisinopril use. Despite appropriate treatment for angioedema during the hospitalization chronicled here, her symptoms did not improve. This led to her care team expanding their differential and exploring other etiologies.

The most common causes of SVC syndrome are malignancies such as non-small-cell lung cancer and mediastinal non-Hodgkin’s lymphoma [4]. Thrombotic occlusion of the SVC in the setting of indwelling venous catheters and cardiac devices has become an increasingly common presentation of SVC syndrome over the last two decades [3,6]. Further history-taking revealed that the patient had no personal or family history of malignancy, nor did she have common risk factors for lung cancer, such as chronic tobacco use or workplace carcinogen exposure [10]. Contrast-enhanced CT scans of the neck and thorax did not show any signs of cancer. However, they did show a thrombus at the cavoatrial junction of the SVC, close to the tip of her indwelling central venous catheter port. The combination of symptoms and imaging findings led to the final diagnosis of SVC syndrome.

Treatment of SVC syndrome depends entirely on the cause, as malignant etiologies may require chemotherapy, radiation, anticoagulation, and endovascular procedures, whereas benign etiologies typically require endovascular procedures or anticoagulation [4]. Our patient underwent balloon angioplasty of the bilateral brachiocephalic veins and SVC, with subsequent removal of the tunneled catheter port, which led to the rapid resolution of her symptoms. She was also placed on apixaban for six months and aspirin indefinitely to decrease the risk of recurrent venous thrombosis.

## Conclusions

This case report highlights a benign cause of superior vena cava (SVC) syndrome and demonstrates how difficult it can be to differentiate it from other life-threatening conditions. Our patient was initially treated for angioedema due to her face swelling, respiratory distress, and reported history of lisinopril-induced angioedema. However, her inability to respond to traditional angioedema treatments led to the discovery of a port-related thrombosis causing SVC syndrome.
